# Genomic and biologic comparisons of cyprinid herpesvirus 3 strains

**DOI:** 10.1186/s13567-018-0532-z

**Published:** 2018-05-02

**Authors:** Yuan Gao, Nicolás M. Suárez, Gavin S. Wilkie, Chuanfu Dong, Sven Bergmann, Pei-Yu Alison Lee, Andrew J. Davison, Alain F. C. Vanderplasschen, Maxime Boutier

**Affiliations:** 1 Immunology-Vaccinology, Department of Infectious and Parasitic Diseases (B43b), Fundamental and Applied Research for Animals & Health (FARAH), Faculty of Veterinary Medicine, University of Liège, Liège, Belgium; 2 MRC-University of Glasgow Centre for Virus Research, Glasgow, UK; 3 MOE Key Laboratory of Aquatic Food Safety/State Key Laboratory for Bio-control, School of Life Sciences, Sun Yat-sen University, Guangzhou, China; 4 Friedrich-Loeffler Institut, Federal Research Institute for Animal Health, Institute of Infectology, Greifswald-Insel Riems, Germany; 5 Department of Research and Development, GeneReach, Biotechnology Corporation, Taichung, China

## Abstract

Cyprinid herpesvirus 3 (CyHV-3) is the archetypal fish alloherpesvirus and the etiologic agent of a lethal disease in common and koi carp. To date, the genome sequences of only four CyHV-3 isolates have been published, but no comparisons of the biologic properties of these strains have been reported. We have sequenced the genomes of a further seven strains from various geographical sources, and have compared their growth in vitro and virulence in vivo. The major findings were: (i) the existence of the two genetic lineages previously described as European and Asian was confirmed, but inconsistencies between the geographic origin and genotype of some strains were revealed; (ii) potential inter-lineage recombination was detected in one strain, which also suggested the existence of a third, as yet unidentified lineage; (iii) analysis of genetic disruptions led to the identification of non-essential genes and their potential role in virulence; (iv) comparison of the in vitro and in vivo properties of strains belonging to the two lineages revealed that inter-lineage polymorphisms do not contribute to the differences in viral fitness observed; and (v) a negative correlation was observed among strains between viral growth in vitro and virulence in vivo. This study illustrates the importance of coupling genomic and biologic comparisons of viral strains in order to enhance understanding of viral evolution and pathogenesis.

## Introduction

Cyprinid herpesvirus 3 (CyHV-3; genus *Cyprinivirus*, family *Alloherpesviridae*, order *Herpesvirales*), also known as koi herpesvirus (KHV), is the etiologic agent of a lethal disease in common and koi carp (*Cyprinus carpio*) [1]. Since its emergence in the late 1990s, this highly contagious and dreadful disease has spread worldwide [2] and caused severe economic losses [3, 4]. CyHV-3 has been the subject of a growing number of studies and is considered to be the archetypal fish alloherpesvirus [1].

The CyHV-3 genome is 295 kbp in size and thus the largest described among all herpesviruses [5]. The complete DNA sequences of four CyHV-3 isolates derived from various geographical locations have been determined [6, 7]. Analyses of these sequences revealed a high level of genomic identity (> 99%), with inter-strain diversity comprising mainly single nucleotide polymorphisms (SNPs) and minor insertions or deletions (indels). They also indicated the existence of two main genotypic lineages initially termed European (based on one strain isolated in Israel and one strain originating from the USA) and Asian (based one strain isolated in Japan). Low inter-strain diversity and an association of these two lineages with geographic origin were further confirmed by PCR-based partial sequencing of a few variable markers for a substantial number of viral samples [8, 9]. However, preliminary data suggested that the situation might be more complex, as some strains originating from Europe resembled the Asian lineage, and vice versa [6, 8, 10]. Moreover, the coexistence in a single genome of loci belonging to both lineages suggested that recombination may have occurred [11]. However, definitive conclusions regarding these issues are difficult to make, since partial sequencing covering only a few loci was performed.

Many of the sequence differences between CyHV-3 strains concern indels caused by variable number of tandem repeats (VNTRs). VNTR polymorphism has shown some utility for differentiating strains of large DNA viruses, such as human herpesvirus 1 [12]. Analysis of multiple VNTR loci in CyHV-3 provided further support for the existence of the two main lineages described above, and, in addition, 87 haplotypes were identified [13, 14]. The discriminatory power of VNTRs is nonetheless more suited to fine tuning the tips of phylogenetic trees based on non-VNTR polymorphisms (SNPs and indels) [1]. Unfortunately, robust phylogenetic classification of this sort is lacking for CyHV-3 strains due to the low number of complete genome sequences available. This deficiency could be overcome using genome-wide analysis of multiple viral strains by high-throughput sequencing. This approach has been used successfully for several other herpesviruses, including human herpesvirus 1 [15], to give a much fuller picture of strain diversity [16]. Indeed, it was recently applied to sequencing CyHV-3 directly from infected fish tissues, without first isolating viral strains [17]. However, multiple strains were detected in each tissue, potentially causing the derived genome sequences to be artificial composites of the individual strains present.

In our study, we sequenced seven strains of CyHV-3 from various geographical origins, and compared their growth in vitro and virulence in vivo. We confirmed the existence of the two genetic lineages described previously as European and Asian, but identified inconsistencies between the geographic origin and the lineage of few strains. Potential inter-lineage recombinations were also demonstrated, which suggested the existence of a third, as yet unidentified lineage. Analyses of CyHV-3 genes that are disrupted, and therefore non-functional, led to the identification of several non-essential genes, some of which may affect virulence. Finally, a negative correlation was established between viral growth in vitro and virulence in vivo. Overall, our study illustrated the potential of comparing the genomic and biologic characteristics of viral strains in studying viral evolution and pathogenesis.

## Materials and methods

### Cells and viruses

Common carp brain (CCB) cells [18] were cultured in minimum essential medium (Sigma) containing 4.5 g/L glucose (d-glucose monohydrate; Merck) and 10% (v/v) fetal calf serum (FCS). The cells were cultured at 25 °C in a humid atmosphere containing 5% CO_2_. A total of seven CyHV-3 strains from various geographic origins were used (Table 1). The Cavoy strain was amplified from a commercial aliquot of an attenuated vaccine produced by passage in cell culture of an isolate from Israel [19–21]. The T strain was subcloned from a large viral plaque obtained after 30 passages of a Taiwanese isolate on a koi fin cell line [22]. GZ11-SC is a subclone of the GZ11 strain from China [6]. It was generated by transfection of GZ11 DNA into CCB cells, followed by plaque purification of the regenerated virus.

**Table 1:** CyHV-3 strains

Name^a^	Geographic origin	GenBank accession number	References
FL	Belgium	MG925487	[45]
M3	Belgium	MG925490	[46]
I	Israel	MG925489	[46]
Cavoy	Israel	MG925485	[19–21]
E	United Kingdom	MG925486	[47]
T	Taiwan	MG925491	[47]
GZ11-SC	China	MG925488	[6]
U	USA	DQ657948.1	[7]
KHV-I	Israel	DQ177346.1	[7]
J	Japan	AP008984.1	[7]
GZ11	China	KJ627438.1	[6]

^a^ The first seven viral strains listed were used in this study.

### Genetic characterization of CyHV-3 strains

Viral DNA from CyHV-3 strains was characterized by restriction fragment length polymorphism using SacI digestion, and further characterized by full-length genome sequencing using methods described previously [23, 24]. Sequences have been deposited to the GenBank.

### Phylogenetic analysis

A multiple alignment of 11 full-length viral genome sequences was made using MAFFT online version 7 [25]. The sequences included the seven produced in this study and the four available in GenBank for the U, J, KHV-I and GZ11 isolates (Table 1) [6, 7]. The phylogenetic tree was generated using the UPGMA method (unweighted pair-group method with arithmetic means) implemented in the MEGA6 software [26], and evaluated by the interior branch test method with 1000 bootstraps.

### Recombination analysis

Detection of inter-strain recombination, identification of closest parental sequences and localization of possible recombination break points were carried out using the Recombination Detection Program 4 (RDP4) [27]. This software combines a variety of independent methods, including RDP [28], GENECONV [29], BOOTSCAN [30], MaxChi [31], CHIMAERA [32], SISCAN [33] and 3SEQ [34].

### Viral growth curve assay

Triplicate cultures of CCB cells were infected with each CyHV-3 strain at a multiplicity of infection (MOI) of 0.05 plaque-forming unit (pfu)/cell. After an incubation period of 2 h, the cells were washed with phosphate-buffered saline and overlaid with Dulbecco’s modified essential medium (DMEM, Sigma) containing 4.5 g/L glucose and 10% (v/v) FCS. The supernatant was removed from infected cultures at successive intervals (0, 2, 4, 6 and 8 days post-infection (dpi)), and stored at −80 °C. Viral titration was carried out by duplicate plaque assays in CCB cells using methods described previously [35].

### Viral plaque size assay

CCB cells were cultured in six-well plates and inoculated with 200 pfu/well of CyHV-3 for 2 h and overlaid with DMEM containing 4.5 g/L glucose, 10% (v/v) FCS and 1.2% (w/v) carboxymethylcellulose (medium viscosity, Sigma). Viral plaques were treated for indirect immunofluorescent staining by using monoclonal antibody 8G12, which recognizes an unidentified CyHV-3 nuclear protein. Plaques were imaged by using a Nikon A1R confocal microscope, and plaque size was measured by using the ImageJ software [36].

### Fish

Common carp were kept in 60 L tanks at 24 °C. Water parameters were checked twice per week. Microbiological, parasitic and clinical examinations carried out immediately prior to the experiments demonstrated that the fish were healthy. All experiments were preceded by an acclimation period of at least 2 weeks.

### Inoculation of fish

Fish were infected by immersion in constantly aerated water containing 400 pfu/mL of virus for 2 h at room temperature, the volume of water being adjusted to a biomass of approximately 10% according to fish size and number. At the end of the incubation period, the fish were returned to the initial tank.

### Ethics statement

The experiments, maintenance and care of fish complied with the guidelines of the European Convention for the Protection of Vertebrate Animals used for Experimental and other Scientific Purposes (CETS No. 123). The animal studies were approved by the local ethics committee of the University of Liège, Belgium (laboratory accreditation No. 1610008, protocol No. 1059). All efforts were made to minimize animal suffering.

### Statistical analysis

Viral plaque sizes and viral growth curves were compared by two-way ANOVA (analysis of variance) with interactions followed by Bonferroni post hoc test. Survival curves were compared by using Log-rank tests. Correlation analyses were done by Pearson two-tailed r test, and illustrated by linear regression, using Graphpad Prism 5. Statistical analyses of recombination events were generated with the RDP4 software. Calculation of *p* values is based on binomial distribution (RDP), Blast-Like Karlin-Altschul & Permutation (GENECONV), Bootstrapping & binomial distribution & Chi squared test (BOOTSCAN), Chi squared test & permutation (MaxChi, CHIMAERA), Permutation & Z Test (SISCAN) and Exact test (3SEQ). Statistical significance was represented as follows: *ns* not significant; *p < 0.05; **p < 0.01; and ***p < 0.001.

## Results

The goal of our study was to gain insights into the evolution and pathogenesis of CyHV-3 by performing coupled genomic and biologic comparisons. With this goal in mind, seven viral strains were selected (Table 1). These strains originated from various countries and were supposed to represent the European and the Asian lineages of CyHV-3.

### Full-length genome analyses

**Figure 1:**
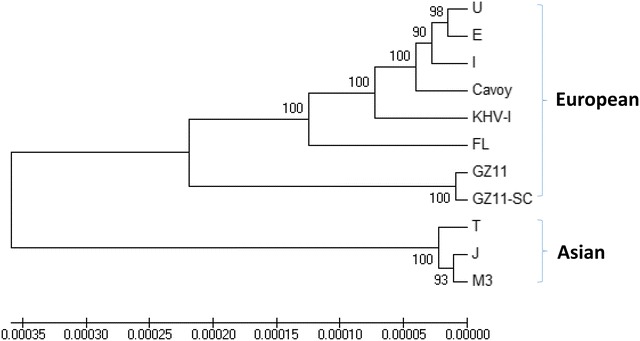
**Phylogenetic analysis of CyHV-3 genome sequences.** The analysis was based on full-length genome sequences excluding one of the terminal direct repeats. The two previously described lineages are indicated. The phylogenetic tree was built using UPGMA in MEGA6 with 1000 replicates. Values on internal branches refer to the percentage of bootstrap replicates in which the branch was found; only values greater than 50% are shown. The scale shows the number of substitutions per nucleotide.

Phylogenetic analysis of the seven new genome sequences and the four published sequences (Table 1) confirmed the high level of similarity (> 99% identity) reported previously. The existence of two major phylogenetic lineages was also confirmed (Figure 1), and a correlation was observed between geographic origin and viral lineage for most strains. However, strain M3 branched in the Asian lineage despite having been isolated in Europe, and strain GZ11 (and its subclone GZ11-SC) had a monophyletic origin with the European lineage despite having been isolated in China. The intermediate position of the GZ11 strain hinted that it may have been generated by recombination between the two lineages. This hypothesis was supported by examination of a genome sequence alignment (data not shown).

### Recombination

**Figure 2:**
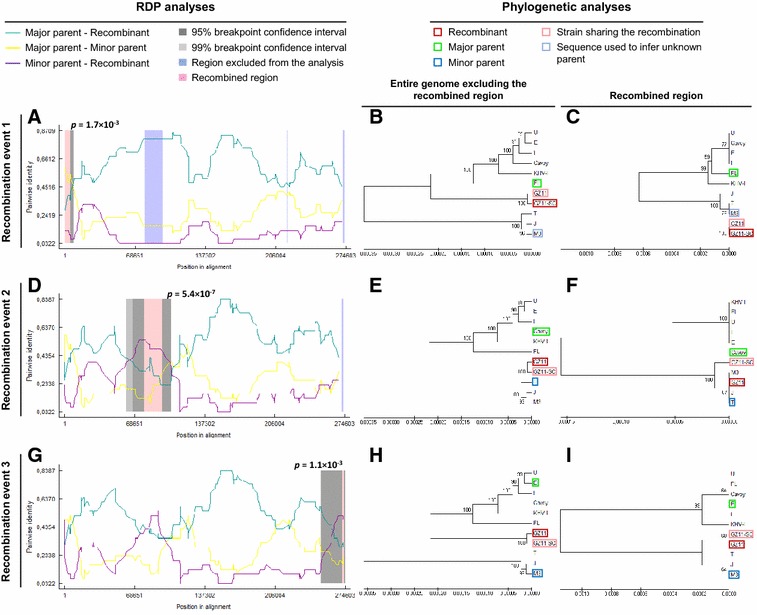
**Recombination between CyHV-3 lineages.** An alignment of the 11 CyHV-3 genomes listed in Table 1 was analysed using the RDP4 software. Three potential recombination events were identified for strains GZ11/GZ11-SC (illustrated by **A**–**I**, respectively). The left part illustrates the results of RDP analyses (**A**, **D** and **G**). The right part presents phylogenetic analyses based on the full-length genome (excluding one of the terminal direct repeats) excluding the region of recombination (**B**, **E** and **H**) or based on the recombination region only (**C**, **F** and **I**) using UPGMA in MEGA6 with 1000 replicates. Values on internal branches refer to the percentage of bootstrap replicates in which the branch was found; only values greater than 50% are shown. The scales illustrate the number of substitutions per nucleotide. The color code used is described at the top.

To explore potential inter-strain recombination, the eleven full-length genomes were analyzed using the RDP4 software. Potential recombination events were detected only in strain GZ11 and its subclone (Table 2 and Figure 2). The results suggested that strain GZ11 consists of a genome in the European lineage (Figures 2B, E and H) in which three recombination events have occurred. One (Figures 2A–C) represented the acquisition of 5.5 kb from the left end of the genome from a third, as yet unidentified lineage (Figure 2C). The other two represented the acquisition of 17.5 and 1.9 kb, respectively, from the Asian lineage (Figures 2F and I). Recombination events 1 and 2 were predicted by 6 detection methods suggesting a high probability of occurrence. In contrast, recombination event 3 was supported by only one detection method and should therefore be treated with caution.

**Table 2:** Recombination events in strain GZ11

Event	Genome region^a^	Major parent^b^	Minor parent^c^	Detection method^d^						
				R	G	B	M	C	S	T
1	117-5598	FL	Unknown	**	*	*	**	*	ns	*
2	77366-94864	Cavoy	T	***	***	***	***	***	ns	***
3	269851-271724	E	M3	**	ns	ns	ns	ns	ns	ns

^a^Coordinates are listed in relation to the sequence of strain U (GenBank accession number DQ657948.1). The results for strain GZ11-SC were the same as for strain GZ11.

^b^Major parent strain was automatically predicted by the RDP software. It represents the closest relative of the recombinant strain taking into account the entire genome but excluding the recombination region.

^c^Minor parent was automatically predicted by the RDP software. It represents the closest relative of the recombinant strain taking into account the recombination region.

^d^Detection methods used in RDP4: R, RDP; G, GENECONV; B, BOOTSCAN; M, MaxChi; C, CHIMAERA; S, SISCAN; T, 3SEQ. Statistical significance is indicated according to the code described in "Materials and methods".

### Viral growth in vitro

**Figure 3:**
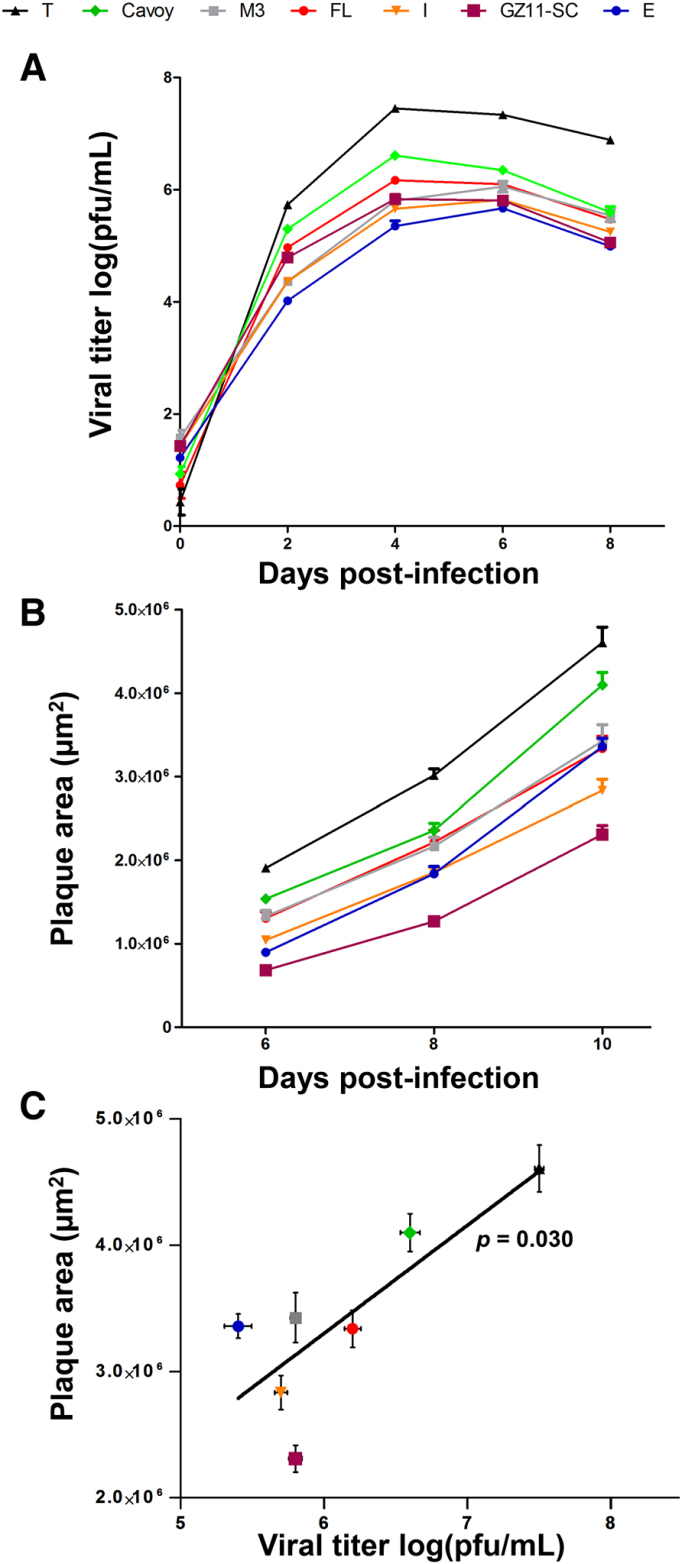
**Comparisons of viral growth in vitro.** **A** Viral growth assay. CCB cells were infected with the strains indicated (see top of the figure for the symbol code used) and the log10 value of the titer (pfu/mL) in the cell supernatant was determined at the indicated dpi. Data are presented as the mean + SEM (standard error of the mean) of triplicate measurements. **B** Viral plaque assay. CCB cells were infected with the strains indicated, and plaques areas were measured over time. Data presented are the mean + SEM for measurements of 20 randomly selected plaques. **C** Correlation between plaque size measured at 10 dpi (**B**) and viral titers measured at 4 dpi (**A**). Data presented are the mean ± SEM.

Viral fitness in vitro was assessed by viral growth assay and plaque size assay on CCB cells (Figure 3). All seven tested viral strains grew efficiently, reaching titers of at least 10^5^ pfu/mL at the peak of infection (Figure 3A). The kinetics of viral growth was similar for all strains. The numbers of infectious particles peaked at 4 dpi (strains T, Cavoy, FL and GZ11–SC) or 6 dpi (strains M3, I and E), and then declined due to virion inactivation. Despite these general similarities, statistical analyses at 4 dpi revealed quantitative differences between the strains. For example, strain T (the best-growing strain) reached a titer > 10^7^ pfu/mL, which was > 100 times that of strain E (the worst-growing strain). These analyses revealed that strain T grew more efficiently than all other strains tested (*p* < 0.001), followed by strain Cavoy (*p* < 0.001 vs all other strains), strain FL (*p* < 0.05 vs strain GZ11-SC, *p* < 0.01 vs strain M3, and *p* < 0.001 vs strains I and E), strains M3, I and GZ11-SC (ns among each other, *p* < 0.05 strains I vs E, and *p* < 0.001 strains GZ11-SC and M3 vs strain E), and finally strain E. These results demonstrate that CyHV-3 strains exhibited different abilities to grow in vitro that are unrelated to the lineage to which they belong.

All viral strains tested were capable of forming plaques (Figure 3B). However, the results obtained at 10 dpi revealed significant differences in plaque size between strains. The largest plaques were produced by strain T, and these were about twice as big in area as those observed for strain GZ11-SC, which produced the smallest plaques. Statistical analyses of the results obtained at 10 dpi revealed that strain T produced the largest plaques (*p *< 0.001 vs all strains), followed by strain Cavoy (*p* < 0.001 vs all strains), strains FL, M3 and E (ns between each other, *p* < 0.01 vs strain I, *p* < 0.001 vs strain GZ11-SC), strain I (*p* < 0.01 vs strain GZ11-SC), and finally strain GZ11-SC. The two parameters used to assess viral replication in vitro (virion production in the extracellular medium and plaque size) were positively correlated (*p* < 0.05; Figure 3C).

### Virulence in vivo

**Figure 4:**
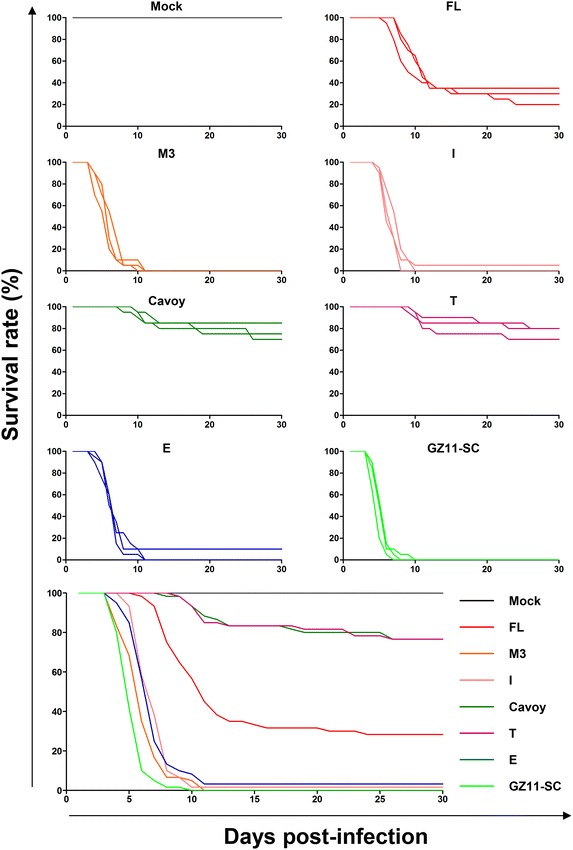
**Comparison of virulence in vivo.** The virulence of the indicated strains was tested in carp (triplicate groups each consisting of 20 subjects, average weight 5.03 ± 3.78 g, 7 months old). On day 0, fish were mock-infected or infected by immersion for 2 h in water containing 400 pfu/mL of virus. Survival rate was measured over a period of 30 dpi. The smaller upper panels show the survival curves observed for replicates. The larger lower panel shows the mean survival curves based on the three replicates.

The levels of virulence of the seven strains were compared after inoculating fish by immersion in water containing the virus, thereby mimicking a natural infection (Figure 4).

Regardless of the viral strain used, all infected fish developed CyHV-3 disease. However, the intensity and the kinetics of appearance of clinical signs varied between strains. Strains Cavoy and T induced the mildest disease, with infected fish expressing folding of the dorsal fin, hyperemia and apathy/anorexia. Around 20% of infected fish succumbed from this infection, including late mortalities induced by neurological symptoms observed in fish surviving the first peak of mortalities. Infection with strain FL led to typical disease including the symptoms described above and associated fin and skin lesions on most fish. Around 70% of infected fish died from this infection. Infection with strains M3, I, E and GZ11-SC led to very acute and highly virulent disease, with almost all fish dying by 10 dpi. None of the mock-infected fish expressed clinical signs or mortality.

Statistical analysis was conducted by two-by-two comparison using a Log-rank test, and the cut-off for significance was adjusted according to the number of comparisons made. Mock-infected groups had higher survival rates compared to all infected fish groups (*p* < 0.0001). Strains Cavoy and T induced higher survival rates than all others strains (ns between these strains, *p* < 0.0001 vs all other strains). Strain FL induced a higher survival rate than strains M3, I, E and GZ11-SC (*p* < 0.0001). Strain GZ11-SC was significantly more virulent than strains I and E (*p* < 0.0001), but not more virulent than strain M3. All other comparisons were not significant.

**Figure 5:**
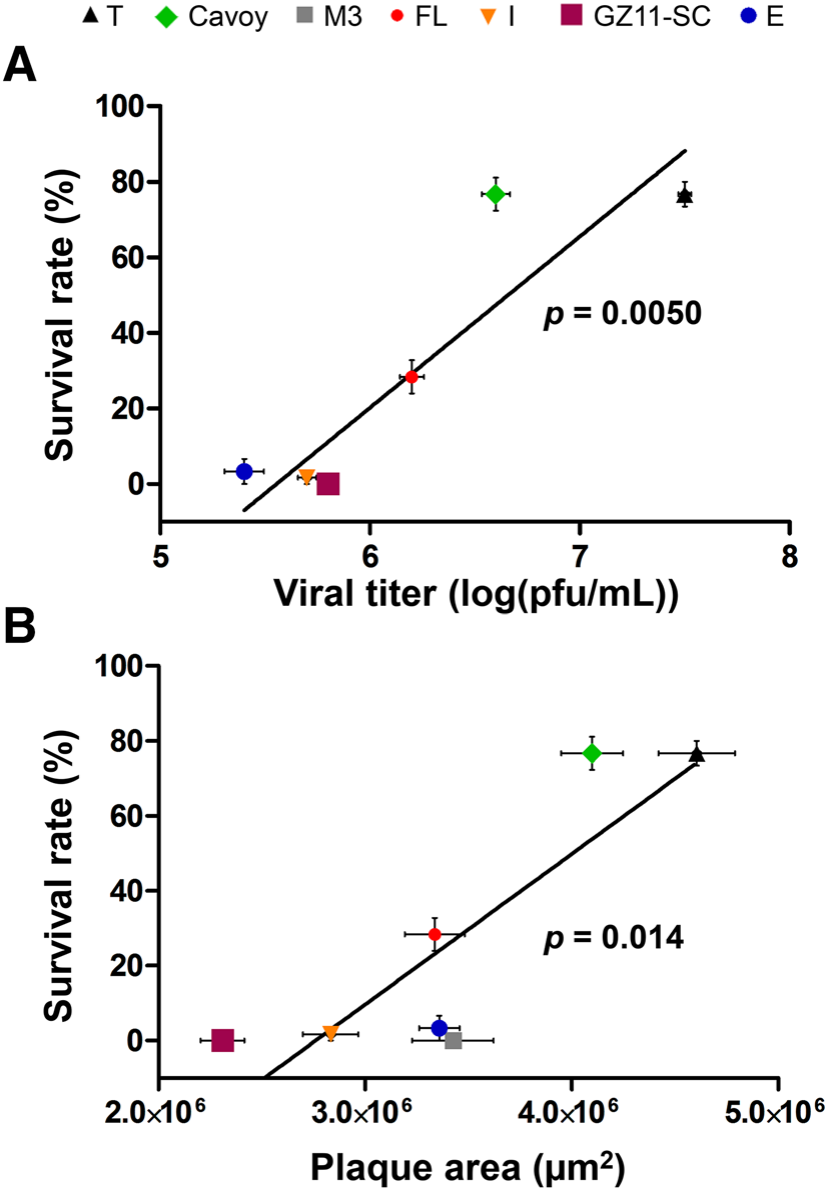
**Correlation of growth in vitro and virulence in vivo.** Data related to viral growth in vitro (viral titer observed 4 dpi and plaque size measured at 10 dpi; see Figure 3) and virulence in vivo (mean survival rates at 30 dpi; see Figure 4) were analysed. **A** Correlation between virulence and viral titer. **B** Correlation between virulence and plaque size. Data presented are the mean ± SEM.

These results indicate that the strains tested can be classified as having low virulence (strains Cavoy and T), moderate virulence (strain FL) and high virulence (strains M3, E, I and GZ11-SC). As observed for viral growth in vitro, there was no clear association between virulence and genetic lineage. Correlation analysis between viral growth in vitro and virulence in vivo was performed as described above. Positive correlations were found between viral replication in vitro (viral growth assay and viral plaque assay) and survival rate in vivo (Figures 5A and B). These results indicate that the adaptation of a viral strain to cell culture is generally associated with its attenuation in vivo.

### Disabled genes

Coupled genomic and biologic comparison of CyHV-3 strains has the potential to indicate biologic roles for genes that are associated with polymorphisms and thus to link them to biological traits. CyHV-3 strains are known to carry mutations (generally frameshifts) in open reading frames (ORFs) that are likely to ablate gene functions, and these mutations may vary from strain to strain. The genes disabled in the strains analysed in our study are summarized in Table 3. Two different situations were noted: ORFs having one or more frameshift near the beginning or in the central part of the ORF that is likely to ablate protein function, and ORFs having a frameshift near the end of the ORF that may not ablate protein function.

**Table 3:** Disrupted genes

Genotype	Strain	ORF^a^																							Virulence^b^
European	U		16		26	27		30		40			55				94		108						NT
	KHV-I		16		26	27		30		40			55						108	116	125	128	139		NT
	GZ11		16		26	27				40			55	57					108						NT
	GZ11-SC	12	16		26					40			55	57					108						High
	E		16		26			30		40			55						108			128			High
	I		16	20	26			30		40			55						108						High
	Cavoy		16		26	27		30		40	48	52	55					105	108					153	Low
	FL		16		26	27	28	30		40			55						108						Moderate
Asian	T				26	27			33	40					64			105							Low
	M3				26					40					64	87		105							High
	J				26					40					64	87		105							NT

^a^Numbers that are struck out identify ORFs having at least one frameshift near the beginning or in the central part of the ORF that is likely to ablate protein function. Numbers that are not struck out represent ORFs having a frameshift near the end of the ORF that may not ablate protein function.

^b^Virulence score based on the results presented in Figure 4. NT not tested.

Analysis of these results together with the biologic properties described above led to several conclusions. First, genes that exhibit mutations incompatible with the expression of a functional protein in any strain that is able to grow in cell culture can be classified as non–essential. These include ORF12, ORF26, ORF27, ORF30, ORF40, ORF52, ORF64, ORF105, ORF128 and ORF153. Second, genes that exhibit mutations incompatible with the expression of a functional protein in a highly virulent strain can be classified as non-essential to virulence. These include ORF12, ORF26, ORF30, ORF40, ORF64, ORF105 and ORF128. Third, mutated ORFs that are found exclusively in low or moderate virulence strains can be classified as potentially important virulence genes. These include ORF27, ORF52 and ORF153.

## Discussion

Comparisons of full-length genome sequences and biological properties in vitro and in vivo for seven viral strains allowed us to draw hypotheses about the evolution of CyHV-3 and the roles of some of its genes.

Analysis of full-length CyHV-3 genome sequences revealed a high level of similarity (> 99% identity), confirmed the existence of two major genetic lineages while also suggesting the existence of a third, as yet undetected lineage, and highlighted the occurrence of inter-lineage recombination. The level of similarity among CyHV-3 strains is by far greater than that reported for other herpesviruses [15, 16] and suggests that the pathogen has emerged relatively recently. This could have been facilitated by the selection for virulence of a viral strain that was already resident in common carp or that had transferred from another host species. However, the existence of identifiable genetic lineages with largely different panoplies of disabled genes, and the single example so far of inter-lineage recombination, suggest that these lineages have been evolving in different host populations. Moreover, the largely consistent association between genetic lineage and geographic origin indicates that the lineages have been spreading independently, with the few inconsistencies and the potential recombination in strain GZ11 (Figure 2 and Table 2) being potentially the consequence of recent geographic spread due to international trading of carp [2]. Importantly, the comparison of the in vitro and in vivo properties of strains belonging to the two lineages did not reveal differences between them, thus implying that inter-lineage polymorphisms do not contribute to the differences in viral fitness observed.

CyHV-3 strains are closely related to each other in sequence, but sufficient differences exist to indicate that their ancestors were infecting various common carp populations long before CyHV-3 disease was first described in the late 1990s. The evolutionary rate of CyHV-3 is unknown, but, if it is similar to that espoused for mammalian alphaherpesviruses (3.5 × 10^−8^ substitutions/nucleotide/year; [37]), the observation that the European and Asian CyHV-3 lineages differ by up to 6 substitutions in the 5130 bp ORF79 encoding the DNA polymerase catalytic subunit would suggest that the virus has been infecting common carp for some tens of thousands of years. On this basis, CyHV-3 disease is more likely to have emerged in the 1990s as an outcome of shifts in host or environmental co-factors rather than as the result of a virus jumping into common carp from another type of cyprinid fish [38]. The ancestral relationship between common carp and CyHV-3 has also potential implications in the Australian plan to use this virus as a biocontrol measure against invading carp [39]. The apparently long association between CyHV-3 and common carp, and the fact that introductions of common and koi carp into Australia have occurred on many occasions since the 1850s, including more recently than the 1990s [40], imply that the presence of CyHV-3 in Australia cannot be ruled out. CyHV-3-associated mass carp deaths in Australia have not been recorded, but this may simply reflect a lack of the environmental co-factors necessary for disease emergence. These considerations have prompted the advice that further assessments of efficacy (not to mention safety) should be carried out before the proposed release is attempted [39, 41].

Our results also identified non-essential viral genes and yielded information on their potential roles in virulence. Some genes are evidently not essential for viral growth in vitro and appear to play no essential role in virulence in vivo. However, the laboratory model of infection used would not have revealed genes that function in modification of host behavior, establishment of latency, reactivation from latency, and excretion of virus and transmission to naïve subjects [23, 38, 42]. Further experiments are needed to extend the in vivo studies.

All of the strains tested, including the Cavoy vaccine strain, were to some degree virulent (Figure 4). This observation implicates that none of the strains lacks the function of an essential virulence gene, such as ORF57 [24]. Interestingly, strain Cavoy expressed a level of virulence comparable to that of strain T, killing 20% of inoculated fish (Figure 4). This residual level of virulence is consistent with earlier observations [21, 43, 44] and could explain why this vaccine was removed from the market soon after its commercialization in the USA. Given that this strain is still used as a vaccine in some countries, its lack of safety must represent a serious source of concern for the aquaculture industry. Disrupted ORFs that are found exclusively in strains of low or moderate virulence are potentially important virulence genes. They include ORF27 (disrupted in strains FL, T and Cavoy), and ORF52 and ORF153 (both disrupted only in strain Cavoy) (Table 2). Notably, the strains with the highest fitness in cell culture were those associated with the longest cell passage history and the lowest virulence in vivo (Figures 3, 4, 5). Although these results suggest that disrupted genes may be potential virulence factors, they are also consistent with gene loss being fortuitously associated with viral adaptation to cell culture. The latter hypothesis is supported by the observation that viral passage in cell culture is frequently associated with the selection of mutations disabling ORF27 [7]. Further experiments are underway to address this issue.

Our study illustrates the power of coupling genomic and biologic comparisons of viral strains to study viral evolution and pathogenesis. It also provides a firm basis for further research on CyHV-3.

## Abbreviations

CCB: common carp brain
CyHV-3: cyprinid herpesvirus 3
DMEM: Dulbecco’s modified essential medium
dpi: days post-infection
FCS: fetal calf serum
indels: insertions or deletions
KHV: koi herpesvirus
ns: not significant
ORF: open reading frame
pfu: plaque-forming unit
RDP: recombination detection program
SEM: standard error of the mean
SNP: single nucleotide polymorphism
UPGMA: unweighted pair-group method with arithmetic means
VNTR: variable number of tandem repeats
